# TG4010 immunotherapy combined with first-line therapy in advanced non-small cell lung cancer (NSCLC): phase IIb results of the TIME study

**DOI:** 10.1186/2051-1426-2-S3-O12

**Published:** 2014-11-06

**Authors:** Elisabeth Quoix, Lecia Sequist, John Nemunaitis, Thaddeus Beck, Piotr Jaskiewicz, Jean-Philippe Oster, Arnaud Scherpereel, Erzsébet Juhász, Zsuzsanna Mark, Rosa Alvarez, Saiama Waqar, Joseph Potz, Nandagopal Vrindavanam, Anton Melnyk, Helen Ross, Jean-Marc Limacher

**Affiliations:** 1 CHRU strasbourg, Strasbourg, France; 2 Massachusetts General Hospital, Cambridge, MA, USA; 3 Mary Crowley Medical Research Center, Dallas, TX, USA; 4 Highlands Oncology Group, Fayetteville, AR, USA; 5 Maria Sklodowska-Curie Memorial Cancer Center and Institute of Oncology - Warsaw, Warsaw, Poland; 6 Centre Hospitalier de Colmar, Colmar, France; 7 CHRU de Lille Hopital Calmette, Lille, France; 8 Orszagos Koranyi TBC es Pulmonologiai Intezet, Budapest, Hungary; 9 Tudogyogyintezet Torokbalint, Torokbalint, Hungary; 10 Hospital Gregorio Marañon, Madrid, Spain; 11 Washington University, St. Louis, WA, USA; 12 Abington Hematology Oncology Associates Inc, Willow Grove, PA, USA; 13 Signal Point Clinical Research Center, Middletown, OH, USA; 14 Texas Oncology, P.A. - Abilene (South), Abilene, TX, USA; 15 Mayo Clinic Arizona, Phoenix, AZ, USA; 16 Transgene S.A., Illkirch Graffenstaden, France

## Background

TG4010 immunotherapy product is a poxvirus (MVA) coding for MUC1 tumor-associated antigen and interleukin-2. A previous study showed that a normal baseline level of Triple Positive Activated Lymphocytes (TrPAL, CD16+CD56+CD69+) might be a predictive biomarker for TG4010 efficacy in advanced NSCLC [1]. TIME is a double-blind Phase IIb/III study comparing the combination of first-line therapy with TG4010 or placebo (NCT01383148).

## Patients and methods

Primary endpoint of the Phase IIb part of the study was to compare progression-free survival (PFS, according to RECIST 1.1) between TG4010 and placebo arms using a Bayesian design, in stage IV NSCLC patients whose tumor express MUC1. Secondary objectives were response rate, safety, survival and subgroup analyses according to stratification factors and level of TrPAL at baseline. A dynamic minimization procedure was applied at randomization for histology, prescription of Bevacizumab, type of chemotherapy, performance status and center.

## Results

217 patients have been enrolled out of which 170 patients with a normal TrPAL level (pre-determined threshold) and an analysis of PFS was conducted in this cohort after 137 events of progression were recorded. The hazard ratio (HR) for PFS is 0.76 (95%CI: 0.54-1.06). This corresponds to a 97.5% Bayesian probability that the true HR is <1, passing the threshold of 95% needed to consider the endpoint met in patients with normal TrPAL. TG4010 related adverse events were limited to mild or moderate fever and injection site reaction. Analysis in the 75% of patients with the lowest baseline level of TrPAL (three lowest quartiles, n = 152) shows a HR for PFS of 0.72 (95%CI: 0.50-1.03) consistent with the observation made in the previous study. Additional pre-planned analyses by subgroup show that patients with non-squamous tumors had a statistically significant improvement in PFS when treated with TG4010 (n = 145, HR = 0.67; CI: 0.46-0.97; p = 0.016) and especially when belonging to the three lowest quartiles (n = 131, HR = 0.63; 95% CI: 0.42-0.93, p = 0.009). Overall survival data will be presented at the time of the meeting.

## Conclusion

These data confirm TG4010 efficacy and safety profile in stage IV NSCLC especially in patients with low level of TrPAL before treatment. They warrant the continuation of the TIME study with its Phase III part.
